# Sex Differences in the Relation between Comorbidities and Prognosis in Hospitalized Patients with COVID-19

**DOI:** 10.1155/2022/8267056

**Published:** 2022-08-18

**Authors:** Noushin Mohammadifard, Fahimeh Haghighatdoost, Maryam Nasirian, Parisa Zakeri, Kamal Heidari, Shaghayegh Haghjooy Javanmard, Nizal Sarrafzadegan

**Affiliations:** ^1^Hypertension Research Center, Cardiovascular Research Institute, Isfahan University of Medical Sciences, Isfahan, Iran; ^2^Interventional Cardiology Research Center, Cardiovascular Research Institute, Isfahan University of Medical Sciences, Isfahan, Iran; ^3^Epidemiology and Biostatistics Department, Health School, Isfahan University of Medical Sciences, Isfahan, Iran; ^4^Heart Failure Research Center, Cardiovascular Research Institute, Isfahan University of Medical Sciences, Isfahan, Iran; ^5^Social Determinants of Health Research Center, Isfahan University of Medical Sciences, Isfahan, Iran; ^6^Applied Physiology Research Center, Isfahan Cardiovascular Research Institute, Isfahan University of Medical Sciences, Isfahan, Iran; ^7^Isfahan Cardiovascular Research Center, Cardiovascular Research Institute, Isfahan University of Medical Sciences, Isfahan, Iran; ^8^School of Population and Public Health-Faculty of Medicine, University of British Columbia, Vancouver, Canada

## Abstract

**Purpose:**

There is a lack of information of the difference in sex-aggregated prevalence of comorbid noncommunicable disease (NCD) in patients hospitalized with COVID-19 in Iran. This study aimed to evaluate sex differences in the relation between medical comorbidities and subsequent death in patients hospitalized with COVID-19.

**Methods:**

All subsequently hospitalized patients with a diagnosis of moderate to severe COVID-19 since February 19^th^ to June 14^th^, 2020, in Isfahan, Iran, were recruited in the ongoing I-CORE Registry. Real-time reverse-transcription polymerase chain reaction (RT-PCR) testing was done upon admission. Data on preexisting comorbid NCDs including hypertension, coronary heart disease (CHD), diabetes mellitus (DM), cancers, chronic renal disease (CRD), and chronic respiratory disease were collected through self-reported questionnaires.

**Results:**

Overall, 12,620 individuals were enrolled in this registry of which 4,356 were positive for the COVID-19 RT-PCR test. In the whole population, in women, DM, hypertension, and CHD, and in men, DM, CHD, and hypertension were, respectively, the most frequent comorbidities. The frequency of at least one NCD did not differ between men and women, but a greater proportion of women had two or more NCDs. Increasing the number of comorbidities was associated with higher death frequency and mortality risk in the unadjusted model but remained no longer significant after adjustment for age. There was no statistically significant difference in this regard between men and women.

**Conclusion:**

Overall, we found that DM, hypertension, and CHD were the most frequent comorbidities. Although comorbidities were more frequent among women, mortality risk did not significantly differ between men and women.

## 1. Introduction

A novel coronavirus emerged in December 2019 in Wuhan, China, and now is spreading rapidly worldwide [1]. According to the World Health Organization (WHO) announcement, it became a pandemic on March 11, 2020 [2]. Sex difference exists in the prevalence and/or prognosis of infectious diseases, including COVID-19 which might be attributed to their differences in immune responses as well as cultural and behavioral factors [3]. However, this matter has been predominantly neglected in epidemiological studies [3, 4].

Global health 50/50, using a large survey, has implied that men affected by COVID-19 are at greater risk of mortality compared with women [5]. However, a meta-analysis of 206,128 reported cases suggested that in spite of a similar proportion of confirmed cases between sexes, men were more likely to require intensive treatment unit admission or die [4]. These outcomes might be attributable to a more robust inherent and a better adaptive antiviral response in women compared with men or to the medical history of patients. In addition, middle-aged and elderly individuals with noncommunicable diseases (NCDs) such as cardiovascular diseases (CVD), hypertension, and diabetes mellitus (DM) are more susceptible to coronavirus and would likely have poorer outcomes [6, 7] as well as increased disease severity [7]. Findings from a systematic review and meta-analysis on 24 articles indicated that hypertension was the most prevalent comorbidity in patients with COVID-19 (20.0%), then DM and CVD with 10.0% and 8.0% prevalence, respectively, and the least prevalent disease was chronic pulmonary disease (3.0%) [7]. Therefore, examining differences in the frequent comorbid NCDs in patients with COVID-19 according to sex will be useful for clinicians to mitigate complications and mortality [7]. Nevertheless, the frequency of comorbidities, according to the patients' sex, has been less addressed in previous reports and is not well-established yet.

In the current study, based on Isfahan COVID-19 Registry (I-CORE) that was established to register all hospitalized COVID-19 patients in Isfahan, Iran, in February 2020 [8], we aim to evaluate sex differences in the relation between medical comorbidities and subsequent death in patients whose COVID-19 RT-PCR test was positive.

## 2. Methods

### 2.1. Design and Participants

The I-CORE registry is an ongoing multicentric registry to recruit all hospitalized patients with COVID-19 in hospitals affiliated to the Medical University of Isfahan (MUI) in Iran [8]. This study was approved by the Ethics Committee of the Research Council of Isfahan University of Medical Sciences (^#^199093). Data used in the current analysis were collected from February 19^th^ to June 14^th^, 2020. All hospitalized patients with suspected COVID-19, regardless of their prognosis or real-time reverse-transcription polymerase chain reaction (RT-PCR) testing results, were enrolled in the I-CORE registry. 12,620 subjects who were admitted to or transferred from other hospitals and health centers to the referral hospitals were recorded. The presence of COVID-19 was diagnosed through the interim guidance of the WHO [9], and moderate to severe cases are usually hospitalized in Iran.

All patients or their immediate families provided written informed consent before recruitment. COVID-19 diagnosis was confirmed by a positive RT-PCR assay [10]; otherwise, they were registered patients with negative RT-PCR. We recruited hospitalized patients with the positive results of the RT-PCR test. Additionally, we excluded those who were recorded as “in bed” (*n* = 623) because their prognosis was not clear and they might bias our results.

### 2.2. Data Collection

Clinical examination was performed by attending specialized physicians on admission and throughout hospitalization. Daily notes on the clinical status of patients along with medical notes recorded by registered nurses were linked to the electronic health information system (HIS). Trained nurses were asked to complete demographic and vital signs questionnaires on admission as well as additional information including date of admission, date of discharge or death, medical history, laboratory tests result, comorbidities, chest X-ray, O_2_ saturation (SO_2_), transfer to intensive care unit (ICU), therapy measures that consist of medicines like (antiviral, antibiotic, corticosteroid, antimalaria, and immune therapy), respiratory support and mechanical ventilation, and clinical outcomes that consist of death or recovery and discharge. To assess comorbidities, participants self-reported the presence of any disease including hypertension, coronary heart disease (CHD), DM, cancer, chronic kidney disease (CKD), and chronic renal disease (CRD). If patients cannot answer the questions, one of their immediate family members responded.

RT-PCR assay was used to detect SARS-CoV-2 RNA based on the WHO protocol from samples of throat swabs [9]. Accordingly, upper respiratory tract specimens were collected from each patient using a nasopharyngeal swab. The swab was inserted from the lower nasal canal to the posterior wall of the nasopharyngeal cavity. Then, it was gently rotated once, and finally, the specimens were dipped into a tube containing 2-3 ml virus preservation solution. Samples were sent to two designated authoritative laboratories. Specimens were tested within 24 hours.

### 2.3. Statistical Analysis

We performed all analyses on the whole population and stratified by sex. We presented continuous variables as means and standard deviation and categorical data as frequencies and percentages. Continuous variables were compared between men and women using an independent sample *t*-test, and the frequency of categorical variables was estimated and compared through the *χ*^2^ test. We also categorized participants into four groups according to the number of comorbidities they had (no comorbidity, one comorbidity, two comorbidities, and three or more comorbidities). To explore the association between the number of comorbidities and mortality risk, a logistic regression model was applied. Odds ratio (OR) and 95% confidence intervals (95% CIs) were estimated in the whole population and stratified by sex. In the whole population, the confounding effect of age (*y*) in model 1 and age and sex in model 2 was adjusted. To check whether the risk of death differs by sex, estimated ORs were compared by the *z*-test. *P* value of less than 0.05 was considered to be statistically significant. Statistical analyses were carried out using STATA software, version 14.

## 3. Results

Table 1 shows the general and demographic characteristics of the study population. We included a total number of 11,997 hospitalized patients with COVID-19 who were discharged or deceased. Of these, 4,356 cases were positive for the COVID-19 RT-PCR test (45.8% women). The mean age was similar between men and women (58.0 ± 18.4 vs. 58.6 ± 18.6 y, respectively). A greater proportion of women had any kind of comorbidities compared with men (43.6% vs. 37.4%). The most prevalent comorbidities in women were DM (20.5%), hypertension (19.6%), and CHD (17.1%), while in men were DM (14.9%), CHD (14.2%), and hypertension (13.8%). The prevalence of these comorbidities was statistically different between men and women (all *P* < 0.0001). There were no significant differences between outcomes occurrence including death as well as recovered and discharged in both sexes.

Table 2 compares the prevalence of comorbidities according to participants' sex and prognosis (recovered and discharged/deceased). While the prevalence of DM (*P* < 0.0001), hypertension (*P* < 0.0001), and CHD (*P*=0.012) was significantly greater among recovered and discharged women compared with counterpart men, and only hypertension was more frequent in deceased women compared with dead men (22.5% vs. 15.7%; *P*=0.027).

The proportion of death according to comorbid NCDs in patients with positive RT-PCR test results according to sex is illustrated in Table 3. In all patients, men and women, the greatest proportion of death was found in the patients with cancer, which was followed by CRD, CKD, CHD, DM, and hypertension, respectively. Comparing men and women revealed no significant differences between death rates in any comorbidity.

Table 4 indicates the association between the number of preexisting comorbidities and the death prognosis. We observed that in all patients with positive RT-PCR, the death rate increased with each additional number of comorbidities (<0.0001). In all patients with three or more comorbid NCDs, 25.7% of patients died, while the corresponding figure in those with no comorbidity was 11.5%. The death rate was similar between men and women in all categories except for those who had one comorbidity (men: 21.9% and women: 15.8%, *P*=0.014).

Table 5 shows the risk of mortality according to the number of preexisting comorbidities in total patients with positive RT-PCR tests and according to sex. Assessing the risk of mortality in the unadjusted model showed that there was a significant association between increasing the number of preexisting comorbidities and the risk of mortality. The mortality risk approximately trebled in patients with three or more NCDs compared with those with no comorbidity (OR (95% CI) 2.67 (1.87–3.81); P for trend <0.0001). This relationship disappeared after adjustment for age in total and both sexes. In the age and sex-adjusted model, mortality risk was marginally higher in patients with three or more comorbidities compared with those with no comorbidity (OR (95% CI): 1.41 (0.97–2.05); *P* for trend = 0.078). There were no significant differences between men and women in ORs of death in unadjusted and age-adjusted models.

## 4. Discussion

In a large sample of hospitalized patients with COVID-19 in Iran, we compared the frequency of underlying comorbid NCDs and mortality rate between men and women. Overall, 15.1% of hospitalized patients died between 19^th^ February and 14^th^ June with no significant difference between men and women (14.5% vs. 15.6%, respectively).Women were more likely to have comorbid NCDs. Mortality risk was associated with increasing the number of preexisting comorbidities. However, these associations vanished after adjustment for age in patients with positive results for the RT-PCR test. Despite the higher prevalence of comorbidities in infected women, their death rate did not differ with men either by the type or by the number of comorbidities.

Sex-imbalance distribution in detected COVID-19 cases has been suggested earlier in some countries [6, 11]; however, when it became an outbreak throughout the world, the Global Health 50/50 research initiative reported conflicting statistics in terms of the numbers of men and women cases [5]. In some countries, the proportion of men and women detected with COVID-19 was similar, but in some others, there was a considerable difference, and the mortality rate constantly remained higher in men [5]. In South Asian countries, around 70% of all COVID-19 cases were men, but they had lower case fatality compared with women [12]. Regarding the duration of hospitalization, results varied across different countries. For instance, in India, no difference was observed, while in Bhutan, women were more probably to be hospitalized for a longer duration [12].

Our findings indicated that a higher prevalence of comorbid NCDs in women was not associated with higher mortality risk when compared with men. In addition, when we categorized patients either by NCDs type or by the number of NCDs, no significant association was observed after adjustment for age in patients with positive results for the RT-PCR test. These results suggest that COVID-19 mortality is independent of comorbidities in both infected men and women that might be explained by the clinical courses of patients. In our earlier study, we observed a significant interaction between NCDs and clinical courses to predict the risk of mortality in COVID-19 patients (revised is in review). Indeed, according to our results, clinical courses play a more crucial role in predicting the risk of death in patients rather than comorbid NCDs, per se. In a recent retrospective study on adults hospitalized for COVID-19, despite the lack of difference between men and women, a significant interaction for age, sex, and survival, independent of the Charlson comorbidity index, was reported [13]. This study showed that in younger adults (<57 y), male sex decreased COVID-19 prognosis by 50%, while in older males, it increased by around 20% [13]. On the other hand, differences between males and females may be mediated by age. However, due to the low frequency of comorbidities in our study population, we were not able to examine this association when participants were stratified into 10 y categories of age.

Irrespective of sex, the presence of some comorbidities like hypertension, CHD, or DM is associated with higher severity of COVID-19 and increases the risk of hospitalization and mortality [14–17]. Consistently, we observed that compared with patients with no NCDs, the presence of comorbidities was associated with a greater risk of mortality. The mechanisms underpinning the greater risk of death in COVID patients with comorbidities have not been well-established. The impaired immune response caused by chronic metabolic dysfunction, particularly insulin resistance, may explain higher mortality rates in these patients to some extent [18, 19]. “Cytokine storm” as a result of the massive inflammatory response induced by coronavirus infection can potentially destroy local and systematic tissues and decrease lymphocyte count. Therefore, chronic inflammation in cases with comorbidities may be one of the reasons explaining higher disease severity and mortality. Moreover, reduced heat shock response (HSR), as an anti-inflammatory pathway, in patients with metabolic diseases can lead to impaired inflammation resolution and enable the COVID-19 virus to be amplified and propagated through all tissues [20].

Unlike some earlier studies [6, 11], we found no significant differences in the risk of COVID-19 mortality between men and women. Several factors may explain the poorer prognosis in men. For instance, women have more potent inflammatory, antiviral, and humoral immune responses than men during viral infections [3, 21], which may result in better clearance of viruses, as well as augmented tissue damage at later stages of the viral disease [22]. In contrast, men have higher levels of angiotensin-converting enzyme 2 (ACE2), which is a well-known receptor of SARS-CoV-2, and the cellular serine protease TMPRSS2 which is necessary for COVID-19 entry into target cells [11]. On the other hand, some contributors may alleviate the prognosis of COVID-19, such as access to the healthcare system at an opportune time, clinical manifestations of COVID-19 on the cardiovascular system, and socioeconomic status [23]. In addition, either sex which means biological differences or gender which refers to sociocultural and behavioral differences could play an essential role in COVID-19 patients' outcomes [24]. For instance, differences between men and women in terms of rejection of health and social recommendations such as social isolation, social, distance, social obligations, stressful life events, low quality of life, and low socioeconomic status among COVID-19 might be other plausible reasons for sex-based differences in fatality rate [23].

### 4.1. Strengths and Limitations

Our study comes with several strengths. To our knowledge, this is the first report comparing the prevalence and death related to comorbidities between men and women hospitalized with COVID-19. Our study sample consisted of a large number of patients who underwent RT-PCR from different socioeconomic statuses which can affect their health behaviors and has good generalizability. However, this study has its limitations. First, the information about comorbidities was self-reported which may be biased. Second, RT-PCR has low sensitivity, and therefore, it is possible that our findings be under or overestimated. Third, due to lack of information on several confounders such as socioeconomic status and lifestyle factors, we were not able to control their effects. Finally, this is a cross-sectional analysis that provides descriptive associations based on our registry, while further studies with longitudinal prospective design are needed to confirm the causality.

### 4.2. Perspectives and Significance

The death rate increased by the number of preexisting comorbid NCDs with no difference among men and women. Although women with COVID-19 had more NCDs, mortality risk did not differ between men and women. Further prospective longitudinal studies with more emphasis on the sex-specific association are warranted to confirm these findings.

## Figures and Tables

**Table 1:** Basic characteristics and comorbidities frequency of hospitalized patients with positive real-time reverse-transcription polymerase chain reaction test results for COVID-19.

Characteristics	Total	Women	Men	*P* value^1^
Number of participants	4,356 (36.3)	1,996 (45.8)	2,360 (54.2)	—

Age (mean, SD), y	58.4 (18.5)	58.6 (18.6)	58.0 (18.4)	0.284

Underlying comorbidities				
DM	761 (17.5)	409 (20.5)	352 (14.9)	<0.0001
Hypertension	717 (16.5)	391 (19.6)	326 (13.8)	<0.0001
CHD	675 (15.5)	341 (17.1)	334 (14.2)	0.008
CRD	276 (6.3)	140 (7.0)	136 (5.8)	0.091
Cancer	116 (2.7)	54 (2.7)	62 (2.6)	0.873
CKD	157 (3.6)	66 (3.3)	91 (3.9)	0.332
Any NCD	1752 (40.2)	870 (43.6)	882 (37.4)	<0.0001

Clinical outcome				
Death, *n* (%)	658 (15.1)	289 (14.5)	369 (15.6)	0.288
Recovered/discharge, *n* (%)	3698 (84.9)	1707 (85.5)	1991 (84.4)	0.288

^1^ *P* value between men and women in positive RT-PCR. Values are numbers and percentages otherwise indicated. RT-PCR, real-time reverse-transcription polymerase chain reaction; SD, standard deviation; CHD, coronary heart disease; CKD, chronic kidney disease; DM, diabetes mellitus; CRD, chronic respiratory disease; NCD, noncommunicable disease.

**Table 2:** Frequency of comorbidities in discharged and deceased hospitalized patients with positive real-time reverse-transcription polymerase chain reaction test results for COVID-19.

Discharged	Women (*n* = 1,707)	Men (*n* = 1,991)	*P* value
DM	332(19.4)	274(13.8)	<0.0001
Hypertension	326(19.1)	268(13.5)	<0.0001
CHD	269(15.8)	256(12.9)	0.012
CRD	102(6.0)	102(5.1)	0.258
Cancer	37(2.2)	40(2.0)	0.736
CKD	51(3.0)	67(3.4)	0.515
Deceased	Women **(*n*** **=** **289)**	Men **(*n*** **=** **369)**	
DM	77(26.6)	78(21.1)	0.099
CHD	72(24.9)	78(21.1)	0.252
Hypertension	65(22.5)	58(15.7)	0.027
CRD	38(13.1)	34(9.2)	0.109
Cancer	17(5.9)	22(6.0)	0.966
CKD	15(5.2)	24(6.5)	0.479

RT-PCR, real-time reverse-transcription polymerase chain reaction; CHD, coronary heart disease; DM, diabetes mellitus; CRD, chronic respiratory disease; CKD, chronic kidney disease.

**Table 3:** Death rate according to the type of comorbidities and sex in hospitalized patients with positive real-time reverse-transcription polymerase chain reaction test results for COVID-19.

	Total, *n* (%)	Women, *n* (%)	Men, *n* (%)	*P* value^1^
N (%)	658 (15.1)	289 (14.5)	369 (15.6)	—
DM	155 (20.4)	77 (18.8)	78 (22.2)	0.255
CHD	150 (22.2)	72 (21.1)	78 (23.4)	0.484
Hypertension	123 (17.2)	65 (16.6)	58 (17.8)	0.680
CRD	72 (26.1)	38 (27.1)	34 (25.0)	0.685
Cancer	39 (33.6)	17 (31.5)	22 (35.5)	0.649
CKD	39 (24.8)	15 (22.7)	24 (26.4)	0.602

CHD, coronary heart disease; DM, diabetes mellitus; CRD, chronic respiratory disease; CKD, chronic kidney disease. ^$^About 5% of COVID-19 patients have been hospitalized by the time of study (men: 5.6%; women: 4.6%). ^*∗*^*P* value comparing death rate in men and women.

**Table 4:** Death rate according to the number of comorbidities and sex in hospitalized patients with positive real-time reverse-transcription polymerase chain reaction test results for COVID-19.

	No comorbidity	One	Two	Three and more	*P* value
*n* (%)	2,604 (59.8)	1,004 (23.0)	569 (13.1)	179 (4.1)	<0.0001
Death	299 (11.5)	192 (19.1)	121 (21.3)	46 (25.7)	
Men, *n* (%)	1,529 (62.2)	570 (23.2)	280 (11.4)	81 (3.3)	<0.0001
Death	173 (11.7)	120 (21.9)	56 (21.6)	20 (26.7)	
Women, *n* (%)	1179 (56.2)	485 (23.1)	322 (15.3)	113 (5.4)	<0.0001
Death	126 (11.2)	72 (15.8)	65 (21.0)	26 (25.0)	
*P* value^*∗*^	0.683	0.014	0.849	0.801	

**Table 5:** Odds ratio and 95% confidence intervals of mortality according to the number of preexisting comorbidities in hospitalized patients with positive real-time reverse-transcription polymerase chain reaction test results for COVID-19.

Number of comorbidities	OR (95% CI) for death				
	No comorbidity	One	Two	Three and more	*P* value
Crude	1	1.82 (1.49–2.22)	2.08 (1.65–2.63)	2.67 (1.87–3.81)	<0.0001

Age-adjusted	1	1.11 (0.90–1.38)	1.10 (0.86–1.41)	1.38 (0.95–2.00)	0.108

Sex and age-adjusted	1	1.12 (0.90–1.38)	1.12 (0.87–1.44)	1.41 (0.97–2.05)	0.078

Men					
Crude	1	2.11^1^ (1.63–2.73)	2.08^2^ (1.49–2.91)	2.74^3^ (1.60–4.69)	<0.0001
Age-adjusted	1	1.40^4^ (1.07–1.84)	1.16^5^ (0.82–1.65)	1.43^6^ (0.82–2.49)	0.086

Women					
Crude	1	1.49^1^ (1.09–2.03)	2.11^2^ (1.51–2.93)	2.65^3^ (1.63–4.28)	<0.0001
Age-adjusted	1	0.80^4^ (0.57–1.13)	1.03^5^ (0.72–1.46)	1.32^6^ (0.79–2.19)	0.467

Comorbidities include coronary heart disease, chronic kidney disease, diabetes mellitus, cancer, and chronic respiratory disease. OR (95% CI), odds ratio (95% confidence interval). ^1^*P* value for comparing OR in men with women, estimated by the *z*-test = 0.087. ^2^*P* value for comparing OR in men with women, estimated by the *z*-test = 0.961. ^3^*P* value for comparing OR in men with women, estimated by the *z*-test = 0.921. ^4^*P* value for comparing OR in men with women, estimated by the *z*-test = 0.012. ^5^*P* value for comparing OR in men with women, estimated by the *z*-test = 0.623. ^6^*P* value for comparing OR in men with women, estimated by the *z*-test = 0.831.

## Data Availability

The datasets generated and/or analyzed during the current study are not publicly available. The dataset of I-CORE is confidential, and care needs to be taken to avoid deductive disclosure. The data are available from the corresponding author upon request.
